# Thymoquinone ameliorates diabetic phenotype in Diet-Induced Obesity mice via activation of SIRT-1-dependent pathways

**DOI:** 10.1371/journal.pone.0185374

**Published:** 2017-09-26

**Authors:** Shpetim Karandrea, Huquan Yin, Xiaomei Liang, Angela L. Slitt, Emma A. Heart

**Affiliations:** 1 Department of Molecular Pharmacology and Physiology, University of South Florida, Tampa, Florida, United States of America; 2 Department of Pharmaceutical Sciences, University of Rhode Island, Kingston, Rhode Island, United States of America; Universidad Pablo de Olavide, SPAIN

## Abstract

Thymoquinone, a natural occurring quinone and the main bioactive component of plant *Nigella sativa*, undergoes intracellular redox cycling and re-oxidizes NADH to NAD^+^. TQ administration (20 mg/kg/bw/day) to the Diet-Induced Obesity (DIO) mice reduced their diabetic phenotype by decreasing fasting blood glucose and fasting insulin levels, and improved glucose tolerance and insulin sensitivity as evaluated by oral glucose and insulin tolerance tests (OGTT and ITT). Furthermore, TQ decreased serum cholesterol levels and liver triglycerides, increased protein expression of phosphorylated Akt, decreased serum levels of inflammatory markers resistin and MCP-1, and decreased NADH/NAD^+^ ratio. These changes were paralleled by an increase in phosphorylated SIRT-1 and AMPKα in liver and phosphorylated SIRT-1 in skeletal muscle. TQ also increased insulin sensitivity in insulin-resistant HepG2 cells via a SIRT-1-dependent mechanism. These findings are consistent with the TQ-dependent re-oxidation of NADH to NAD^+^, which stimulates glucose and fatty acid oxidation and activation of SIRT-1-dependent pathways. Taken together, these results demonstrate that TQ ameliorates the diabetic phenotype in the DIO mouse model of type 2 diabetes.

## Introduction

Maintenance of glucose homeostasis involves insulin secretion from the pancreatic β-cells in response to a rise in blood glucose, and insulin action in target tissues (predominantly liver, muscle, and adipose tissue) to stimulate glucose entry and utilization, and inhibit hepatic glucose production [1]. Development of type 2 diabetes (T2D) involves both peripheral insulin resistance and pancreatic β-cell dysfunction. Insulin resistance, the inability of peripheral tissues to properly respond to insulin, is initially compensated by a rise in insulin output in order to maintain normoglycemia [1]. However, this compensatory mechanism is impaired in individuals predisposed to T2D, and later results in overt hyperglycemia [2, 3].

Thymoquinone (TQ) is the main bioactive component of *Nigella sativa*, a spice plant of *Ranunculacea* family, and a traditional medicine that has been used to treat diabetes symptoms and to lower blood glucose [4]. *Nigella sativa* has been reported to increase both insulin secretion and insulin sensitivity [5, 6]. TQ has been shown to reduce hepatic glucose production [7] and protect β-cells from oxidative stress following streptozotocin (STZ) treatment [8]. However, mechanistic studies and comprehensive evaluation of TQ action under physiological diabetic conditions and models is currently lacking.

TQ belongs to the family of quinones, naturally-derived compounds featuring a conjugated double bond system, which is responsible for their reactivity and intracellular process known as “redox cycling” [9]. Our laboratory has been instrumental in establishing the concept that re-oxidation of NADH back to NAD^+^ via quinone-dependent redox cycling lowers cellular reductive poise and facilitates glucose and fatty acid oxidation, and is necessary for the overall health of the cells [9, 10]. Our group has previously shown that TQ supports redox cycling in pancreatic β-cells, resulting in the reduction of NADH/NAD^+^ ratio and normalization of defective glucose-stimulated insulin secretion (GSIS) under chronically elevated glucose via inhibition of acetyl CoA carboxylase (ACC) and enhanced oxidation of glucose and fatty acids [11].

The oxidation status of nicotinamide adenine dinucleotide, represented by the ratio between its reduced and oxidized forms (NADH/NAD^+^) is a critical determinant of the direction of metabolic flux [12, 13], as NAD^+^ promotes oxidative pathways via activation of TCA cycle enzymes [14]. Furthermore, increased intracellular level of NAD^+^ activates SIRT1-dependent metabolic pathways, which stimulate energy metabolism, enhance life span, and can positively regulate insulin secretion and insulin signaling [14, 15].

Here we evaluated the capacity of TQ to ameliorate the diabetic phenotype in a physiologically relevant rodent model of obesity and diabetes, Diet-Induced Obesity (DIO) mice. We hypothesized that sustained decrease in the NADH/NAD^+^ ratio due to TQ-dependent redox cycling will result in the enhanced fuel oxidation and amplification of NAD^+^-dependent SIRT-1 pathway in metabolic tissues, leading to the enhanced insulin sensitivity and improved glucose homeostasis.

## Materials and methods

### Chemicals

Human recombinant insulin, resveratrol, and AICAR were purchased from Tocris Bioscience (Bristol, UK). Nicotinamide was purchased from Acros Organics (Geel, Belgium) and Compound C was purchased from EMD Millipore (Billerica, MA). All other chemicals and reagents were purchased from Sigma (St Louis, MO) unless specified otherwise. Stock solutions of thymoquinone, resveratrol, AICAR, Nicotinamide, and Compound C were prepared in DMSO and added to culture medium to achieve the indicated concentrations.

### Ethics statement

All procedures were performed in accordance with and approved by the Institutional Animal Care and Use Committee (IACUC) of the University of South Florida.

### Animals

Male C57BL/6J mice (6 weeks of age) were purchased from Jackson Laboratories (Bar Harbor, ME) and housed (4 animals per cage) in a USF Animal Facility; room was maintained at a constant temperature (25°C) in a light:dark 12:12-h schedule. Food and water was available *ad libitum*. Body weight was monitored on a weekly basis. Mice were pair fed either control low fat diet, LFD (10% fat cal, Research Diets, New Brunswick, NJ) or high fat diet, HFD (45% fat cal, Research Diets, New Brunswick, NJ). Mice were separated in the following groups: LFD, LFD+TQ, HFD, HFD+TQ. TQ (dissolved in canola oil) was administered daily by oral gavage at 20 mg/kg body weight for the duration of the study. Vehicle only (canola oil) was administered to control groups (LFD and HFD). The dose of TQ was chosen because it was shown to lower blood glucose [16], albeit in a non-physiological rodent model of diabetes. The chosen dose is well below toxic doses established for oral administration in mice [17]. As expected, TQ was well tolerated, and TQ administration did not affect the overall health of the animals in the study. After 24 weeks, animals were euthanized with isoflurane, tissues and serum collected, and either used immediately or were snap frozen in liquid nitrogen and stored in -80°C until further use.

### Cell culture

HepG2 human hepatoma cell line was purchased from American Type Culture Collection (ATCC, Manassas, VA) and cultured in DMEM medium supplemented with 10% FBS, 100 units of penicillin, and 100 μg/mL streptomycin at 37°C in a humified incubator with 5% CO_2_. Cells were made insulin resistant by treatment with 20mM glucose for 18 hours, as previously described [18, 19]. Following high glucose treatment, cells were starved for 2 hours in serum-free medium, prior to treatment with the respective compounds for 24 hours. For inhibitor treatment, cells were pre-incubated with the inhibitors for 30 mins, and the inhibitors were also present during the 24-hour incubation period. To measure insulin signaling, insulin was added during the last 30 minutes. Vehicle-treated cells (0.5% DMSO) in normal (5.5 mM) and high (20 mM) glucose conditions served as controls.

### OGTT and ITT

For *in vivo* studies, animals were anesthetized with ketamine (80 mg/kg body weight). Oral glucose and insulin tolerance tests were performed following a 6 hr fast. Mice were oral gavaged with 2 mg/kg/bw glucose (OGTT), or injected intraperitoneally with 0.5 IU insulin/kg/bw (ITT). Blood glucose, obtained at 0, 15, 30, 60, 90, 120 and 180 minutes from the tail vein was measured with a glucometer (Bayer Contour).

### Cholesterol content

Total cholesterol, HDL, and LDL/VLDL content was determined from serum samples using the HDL and LDL/VLDL Cholesterol Assay Kit (abcam, Cambridge, MA) according to the manufacturer’s protocols.

### Serum profile

Serum levels of insulin, resistin and MCP-1 were determined by Ocean Ridge Biosciences (Deerfield Beach, FL) using a Luminex multiplex protein profiling assay (Luminex Corp., Austin, TX) according to the manufacturer’s protocols.

### Western blot analysis

Liver and soleus muscle tissues were solubilized in RIPA lysis buffer (Pierce, Rockford, IL) using Fast Prep 24G system (MP Biosciences, Santa Ana, CA). After exposure, HepG2 cells were solubilized in RIPA lysis buffer. Protein content was determined using a BCA Protein Assay Kit (Pierce, Rockford, IL) and SDS samples were prepared. Equal amount of protein (100 μg per lane) were electrophoretically separated on SDS-polyacrylamide gel, followed by blotting onto PVDF membrane. Following the transfer, membranes were blocked with TBST (10 mmol/l Tris-HCl pH 7.4, 150 mmol/l NaCl, and 0.1% Tween 20) containing 5% nonfat dry milk (blocking buffer) and incubated with the primary antibodies (diluted in blocking buffer overnight at 4°C) against SIRT-1 (Cell Signaling, cat. #9475), p-SIRT-1 (Cell Signaling, cat. #2314), Akt (Cell Signaling, cat. #9272), p-Akt (Cell Signaling, cat. #9271), AMPKα (Cell Signaling, cat. #5831), p-AMPKα (Cell Signaling, cat. #2535), NQO1 (Santa Cruz, cat. #sc-16464), β-actin (Cell Signaling, cat. #4970), and β-tubulin (Cell Signaling, cat. #2146). Membranes were incubated with goat anti-rabbit immunoglobulin (IgG) secondary antibody (Santa Cruz, cat. #sc-2030) for 1 h at room temperature, and washed 5 times. Proteins were detected by using enhanced chemiluminescence. Semiquantitative analysis of Western blot images were performed using ImageJ.

### Triglyceride content

Triglyceride content was determined in liver and soleus muscle RIPA buffer lysates (lysates as described above) using the Triglyceride kit (Pointe Scientific, Canton, MI) according to the manufacturer’s protocols.

### Metabolomics analysis

Serum levels of glycerol, palmitic acid, oleic acid, and stearic acid were measured by gas chromatography—mass spectrometry (GC/MS) analysis. The GC/MS experiments were performed by the University of Utah Metabolomics Core.

### Determination of nucleotides

NADH/NAD^+^ ratio was determined in liver and soleus muscle using the NAD/NADH assay kit as per the manufacturer’s protocol (Abcam, Cat #65348, Cambridge, UK).

### Quantitative real time RT-PCR

The tissue samples stored in RNAlater (Invitrogen, Carlsbad, CA) were homogenized by using the Fast Prep 24G instrument (MP Biosciences, Santa Ana, CA). Total RNA was prepared using the TRIzol reagent according to the manufacturer's protocol (Invitrogen, Carlsbad, CA) and single-strand cDNA was synthesized from the RNA in a reaction mixture containing optimum blend of oligo(dT) primers and iScript reverse transcriptase (Bio-Rad, Richmond, CA). qRT-PCR amplifications were performed using rEVAlution 2x qPCR Master Mix (Empirical Bioscience, Grand Rapids, MI) in an MyIQ2 Real-Time PCR Detection System (Bio-Rad, Richmond, CA) following manufacturer's protocol. To determine the specificity of amplification, melting curve analysis was applied to all final PCR products. The relative amount of target mRNA was calculated by the comparative threshold cycle method by normalizing target mRNA threshold cycle to those for glyceraldehyde-3-phosphate dehydrogenase (GAPDH). The primers used for analysis were as follows: NQO1: sense primer, 5’-AGGATGGGAGGTACTCGAATC-3’, anti-sense primer, 5’-AGGCGTCCTTCCTTATATGCTA-3’; GAPDH: sense primer, 5’-CTTCACCACCATGGAGAAGGC-3’, anti-sense primer, 5’-GGCATGGACTGTGGTCATGAG-3’.

### Statistical analysis

Data are expressed as means ± SEM. Significance was determined for multiple comparisons using one-way or two-way analysis of variance (ANOVA) followed by Sidak or Holm-Sidak multiple comparisons tests [20, 21] for planned comparisons (as mentioned in each figure) or independent t-test as indicated. A p-value of ≤0.05 was considered significant.

## Results

**Fig 1:**
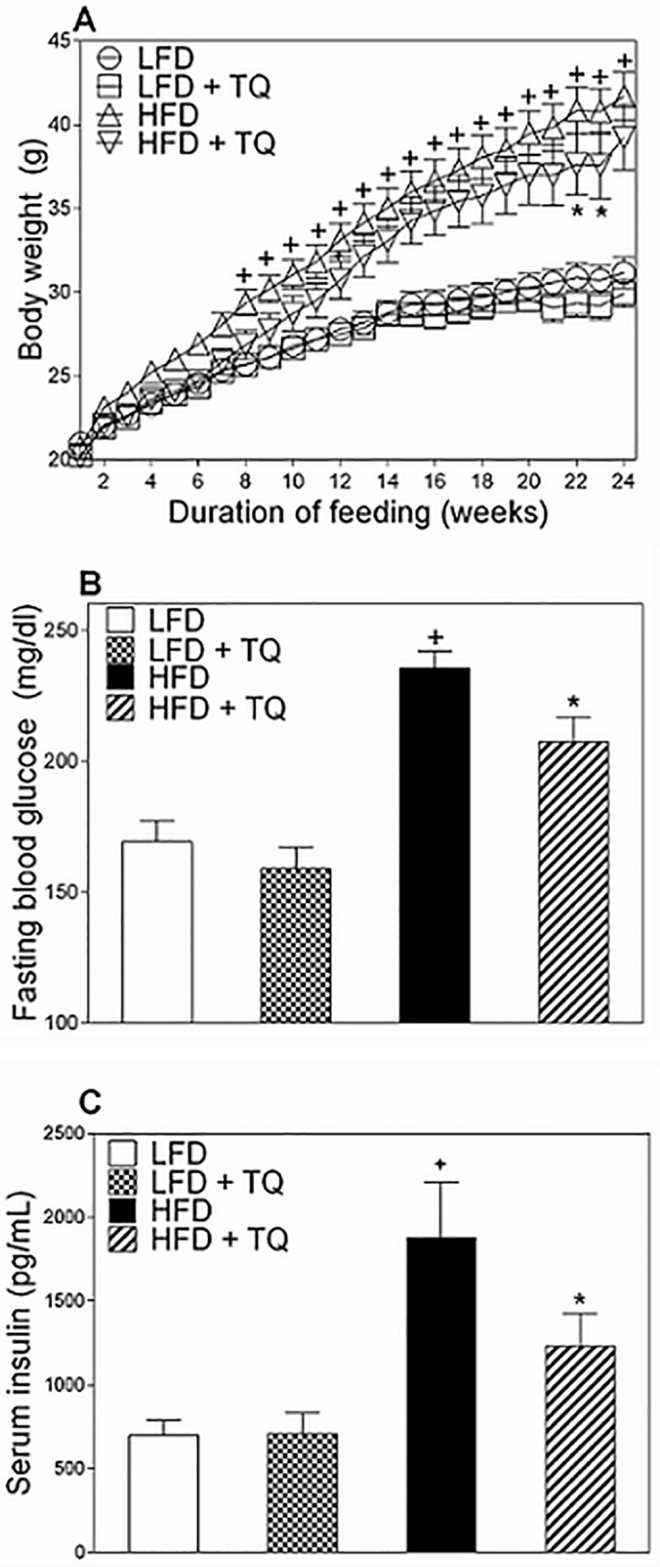
TQ ameliorates weight gain, lowers fasting blood glucose and insulin in DIO mice. (A) Effect of TQ on body weight (B) Effect of TQ treatment on fasting blood glucose after a 6 hour fast. (C) Effect of TQ on serum insulin. Total body weight was measured weekly for the duration of the study. p<0.05 when comparing HFD and LFD (+), and HFD and HFD+TQ (*), using a one-way ANOVA followed by Sidak post-test (A and B) or independent t-test (C). Results are means ± SEM (n = 10–12 mice per treatment group). LFD: low fat diet, HFD: high fat diet, TQ: thymoquinone.

**Fig 2:**
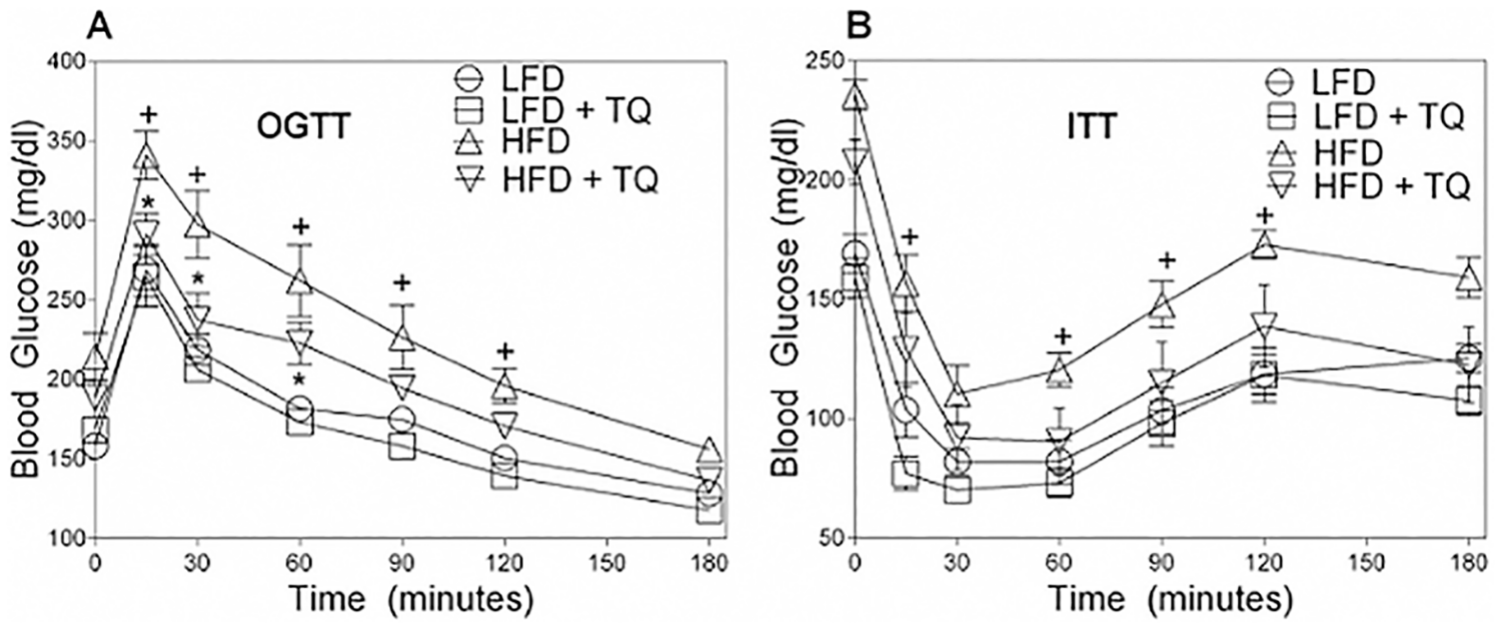
TQ normalizes glucose tolerance and insulin sensitivity. (A) Blood glucose levels in response to oral glucose tolerance test (OGTT). (B) Blood glucose levels in response to insulin tolerance test (ITT). p<0.05 when comparing HFD and LFD (+), and HFD and HFD+TQ (*), using a two-way ANOVA followed by Holm-Sidak post-test. Results are means ± SEM (n = 10–12 mice per treatment group). LFD: low fat diet, HFD: high fat diet, TQ: thymoquinone.

The Diet Induced Obesity (DIO) mice develop obesity, hyperinsulinemia, glucose intolerance and insulin resistance when fed a high fat diet, making them a suitable model to study type 2 diabetes pathophysiology [22, 23]. This is confirmed in our study, where after high fat feeding, mice developed a diabetic phenotype as shown by the weight gain (Fig 1A), elevated fasting blood glucose (BG) and insulin levels (Fig 1B and 1C), and impaired oral glucose and insulin tolerance tests (OGTT an ITT) (Fig 2A and 2B). TQ administration was effective in ameliorating these parameters: TQ lowered body weight (Fig 1A), fasting blood glucose and insulin (Fig 1B and 1C, respectively), and improved glucose tolerance and insulin sensitivity, evaluated by OGTT and ITT (Fig 2A and 2B).

**Fig 3:**
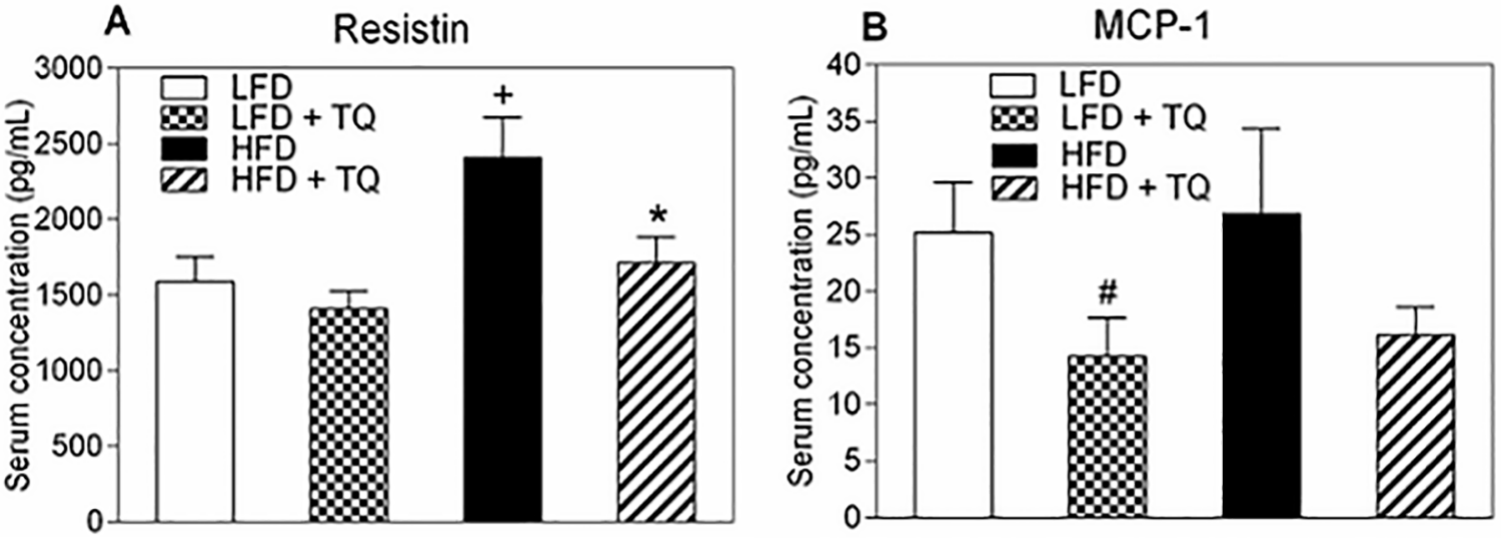
Effects of TQ on serum resistin and MCP-1. (A) Resistin serum concentration. (B) MCP-1 serum concentration. p≤0.05 when comparing (+) HFD and LFD, (*) HFD + TQ and HFD, and (#) LFD and LFD + TQ using independent t-tests. Results are means ± SEM (n = 10–12 mice per treatment group). LFD: low fat diet, HFD: high fat diet, TQ: thymoquinone, MCP-1: monocyte chemotactic protein 1.

Type 2 diabetes is associated with increased inflammation, which can contribute to insulin resistance and is shown to be detrimental to many tissues including pancreatic β-cells [24, 25]. Resistin, a hormone secreted by adipocytes, impairs glucose tolerance and insulin sensitivity in mice [26] and has been associated with insulin resistance in humans [27, 28]. Monocyte chemoattractant protein-1 (MCP-1) is a pro-inflammatory chemokine that can induce insulin resistance [29] and circulating levels of this chemokine are increased in patients with type 2 diabetes [30–32]. TQ lowered serum levels of resistin in DIO mice (Fig 3A). There was a trend to lower the MCP-1 levels, however, this didn’t reach statistical significance in HFD animals (p = 0.06), although TQ decreased MCP-1 in LFD animals (Fig 3B). These results demonstrate the potential of TQ to alleviate tissue inflammation in diabetes and obesity.

**Fig 4:**
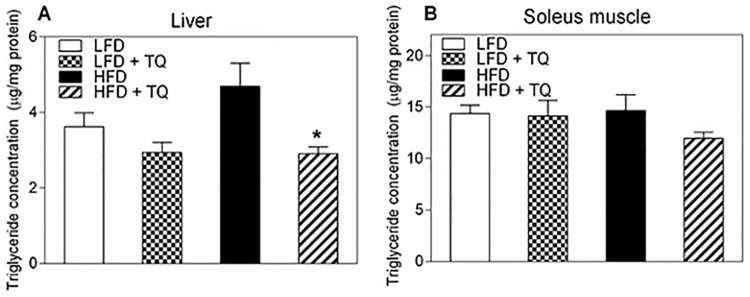
Effects of TQ on triglyceride content in liver and muscle. (A) Triglyceride concentration in liver. (B) Triglyceride concentration in soleus muscle. (*) p<0.05 when comparing HFD + TQ and HFD using a one-way ANOVA followed by Sidak post-test. Results are means ± SEM (n = 8–12 mice per treatment group). LFD: low fat diet, HFD: high fat diet, TQ: thymoquinone.

Elevated levels of triglycerides, together with decreased HDL and increased LDL cholesterol levels are the key identifiers of diabetic dyslipidemia, which can exacerbate insulin resistance [33]. Consistent with our previously reported data demonstrating TQ-dependent increase in fatty acid oxidation [11], and observed increased peripheral insulin sensitivity in this study (as shown by the improvement of the ITT in DIO mice, Fig 2B), TQ ameliorated HFD-dependent increase in liver triglyceride levels (Fig 4A). There was a trend to lower HFD-dependent muscle triglyceride content, however this did not reach statistical significance (Fig 4B). We saw similar trends when analyzed serum glycerol and three relevant fatty acids: palmitic acid, oleic acid, and stearic acid. GC/MS analysis of serum levels of these metabolites were decreased compared to HFD alone (Table 1), however this didn’t reach statistical significance.

**Table 1:** Effect of TQ on serum glycerol and fatty acids.

Metabolite	Treatment			
	LFD	LFD + TQ	HFD	HFD + TQ
Glycerol	844.7 ± 70.1^a^	1282 ± 101.9^c^	1348 ± 124.2^b^	1186 ± 47.1
Palmitic Acid	820.3 ± 26.1	970.4 ± 61.3	862.7 ± 53.8	798.7 ± 23.4
Oleic Acid	2851 ± 179.8	3335 ± 195.8	2807 ± 345.9	2597 ± 114.5
Stearic Acid	381.9 ± 15.0	371.9 ± 22.3	471.2 ± 25.8	437.5 ± 21.4

Results expressed as means ± SEM. n = 10–12 mice/group. Means within the same row with different superscripts differ, p ≤ 0.05 as determined by using a one-way ANOVA followed by Sidak post-test. a, b = LFD vs. HFD only; a, c = LFD vs. LFD + TQ only. TQ = Thymoquinone, LFD = low fat diet, HFD = high fat diet.

**Fig 5:**
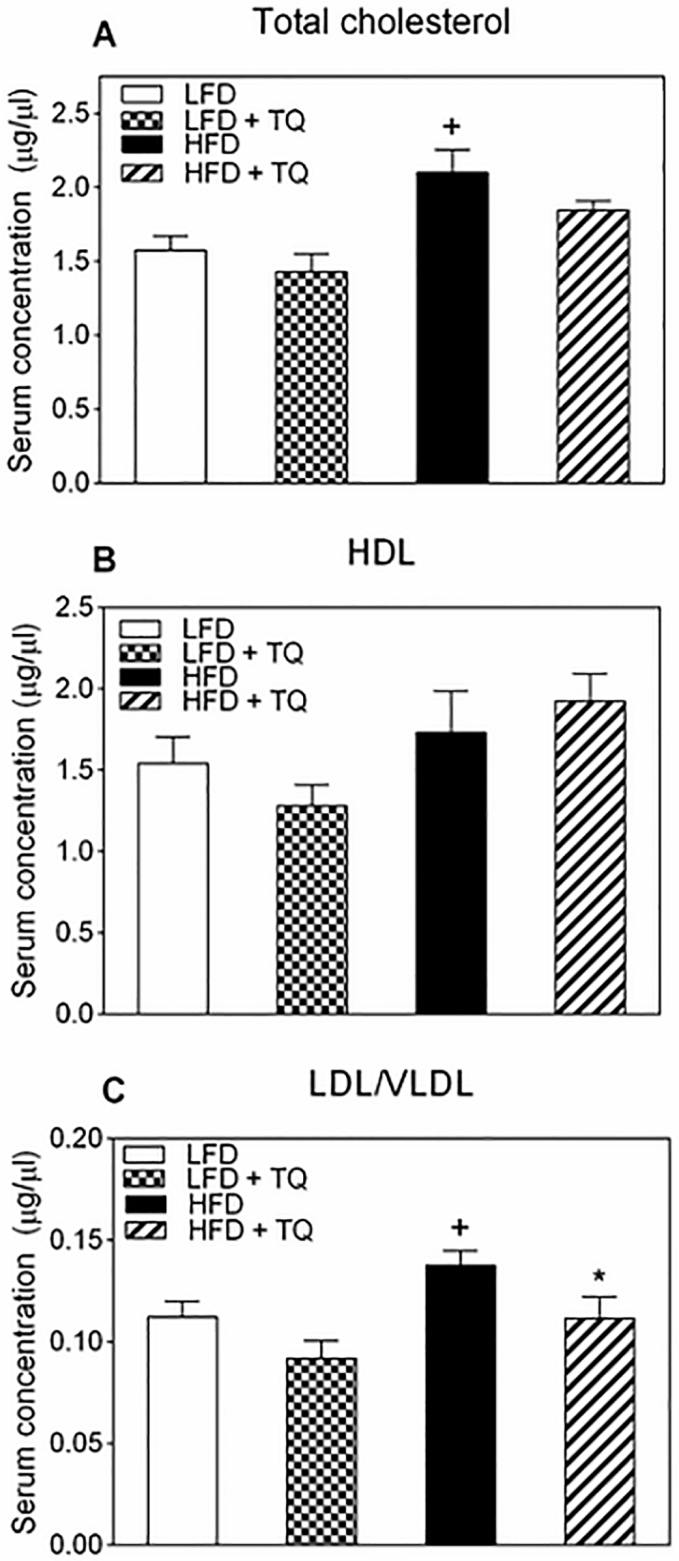
Effects of TQ on serum cholesterol. (A) Total cholesterol serum concentration. (B) HDL cholesterol serum concentration. (C) LDL/VLDL cholesterol serum concentration. p≤0.05 when comparing (+) HFD and LFD, (*) HFD + TQ and HFD using independent t-tests. Results are means ± SEM (n = 6–7 mice per treatment group). LFD: low fat diet, HFD: high fat diet, TQ: thymoquinone, LDL: low-density lipoprotein, HDL: high-density lipoprotein, VLDL: very-low-density lipoprotein.

There was also a trend to decrease serum cholesterol level, albeit statistically not significant (Fig 5A). However, TQ significantly decreased the levels of LDL cholesterol in the serum of HFD animals (Fig 5C), with no effect on the HDL levels (Fig 5B). This effect was selective to the HFD diet, as LFD animals did not demonstrate changes in their HDL or LDL/VLDL cholesterol in response to TQ regimen.

**Fig 6:**
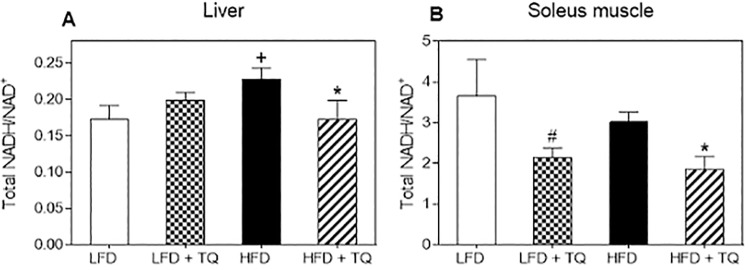
Effects of TQ on NADH/NAD^+^ ratio in liver and soleus muscle. (A) NADH/NAD^+^ ratio in liver. (B) NADH/NAD^+^ ratio in soleus muscle. p ≤ 0.05 when comparing (+) HFD and LFD, (*) HFD + TQ and HFD, and (#) LFD and LFD + TQ using independent t-tests. Results are means ± SEM (n = 8–10 mice per treatment group). LFD: low fat diet, HFD: high fat diet, TQ: thymoquinone.

The lowered tissue triglyceride levels following TQ administration argues for the TQ-dependent activation of the oxidative pathways (and consequent oxidation, rather than deposition of metabolic substrates). NADH/NAD^+^ ratio is important determinant of metabolic flux [14], and our group previously reported that TQ lowers NADH/NAD^+^ ratio in pancreatic β-cells exposed to glucose overload [11]. To confirm that TQ exerts this effect *in vivo*, we measured NADH/NAD^+^ ratio in liver and skeletal muscle. In liver, there was an increase in this ratio in HFD mice (Fig 6A), which is in agreement with prior studies suggesting an increase in NADH in diabetes and obesity [14]. However, we did not observe this change in skeletal muscle (Fig 6B). In both liver and soleus muscle, TQ lowered the NADH/NAD+ ratio in the HFD group compared to HFD alone (Fig 6A and 6B).

**Fig 7:**
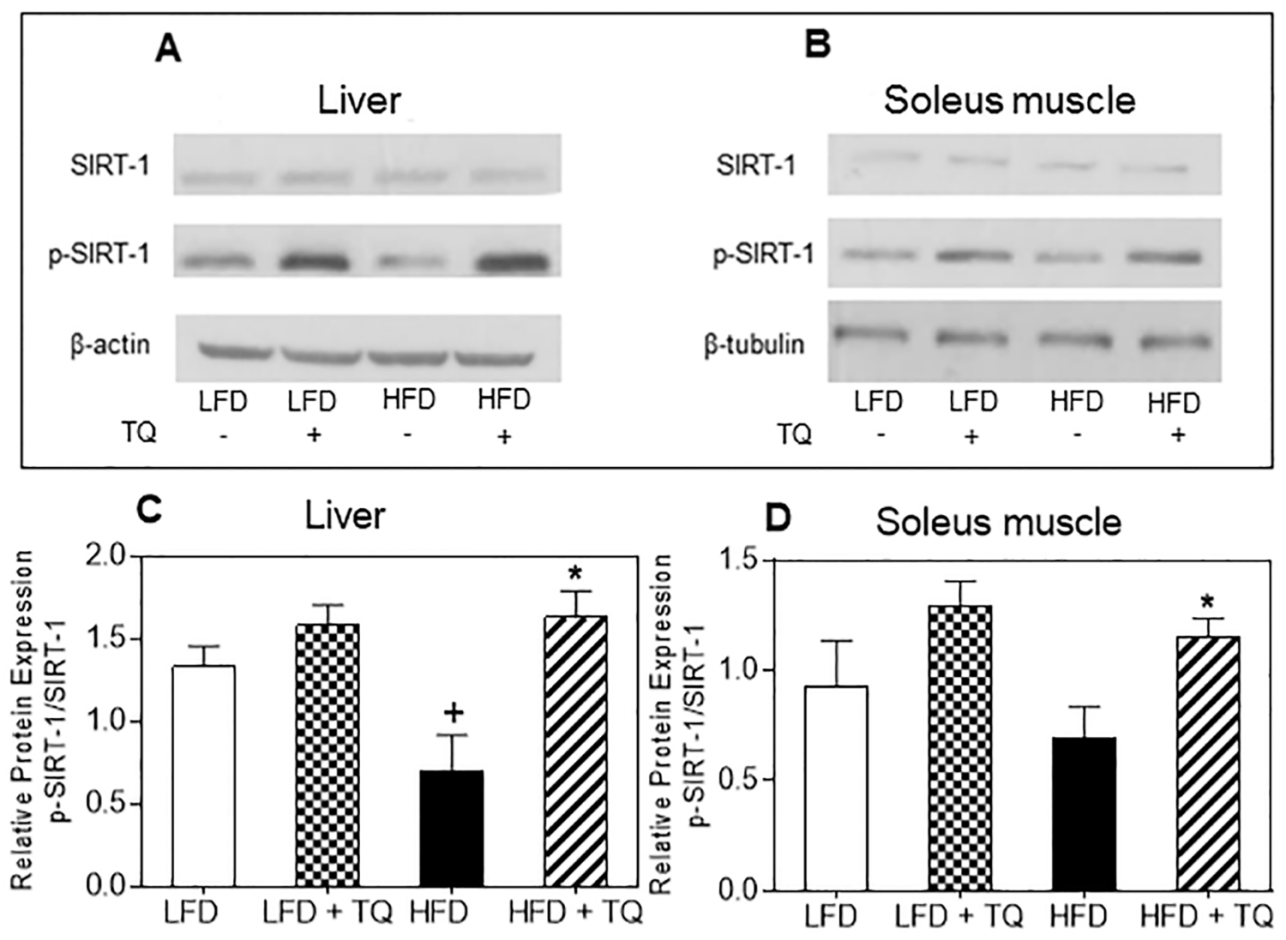
Effects of TQ on SIRT-1 protein expression. (A) Western blot images of SIRT-1 and p-SIRT-1 protein in liver. β-actin was used as a loading control. (B) Western blot images of SIRT-1 and p-SIRT-1 protein in soleus muscle. β-tubulin was used as a loading control. Western blot images are representative of combined liver and soleus muscle lysates from n = 10–12 mice per treatment group. (C and D) Protein band quantification using densitometry from three independent experiments. p ≤ 0.05 when comparing (+) HFD and LFD and (*) HFD + TQ and HFD using independent t-tests LFD: low fat diet, HFD: high fat diet, TQ: thymoquinone.

**Fig 8:**
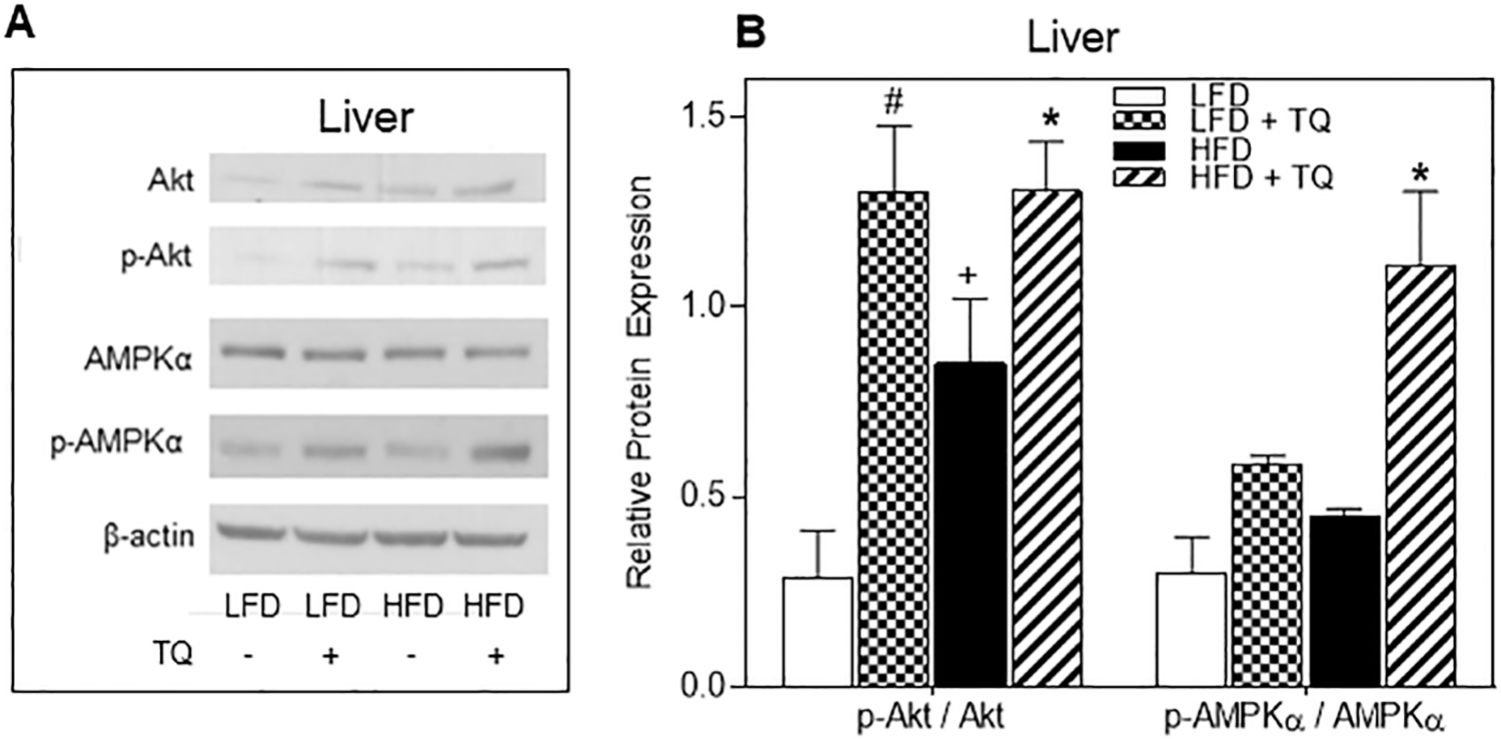
Effects of TQ on Akt and AMPKα protein expression in liver. (A) Western blot images of Akt, p-Akt, AMPKα and p-AMPKα protein in liver. β-actin was used as a loading control. Western blot images are representative of combined liver lysates from n = 10–12 mice per treatment group. (B) Protein band quantification using densitometry from three independent experiments. p≤0.05 when comparing (+) HFD and LFD, (*) HFD + TQ and HFD, and (#) LFD and LFD + TQ using independent t-tests. LFD: low fat diet, HFD: high fat diet, TQ: thymoquinone.

Since NADH/NAD^+^ ratio is known to regulate SIRT-1 pathway, we analyzed effect of TQ feeding on this pathway in the liver and soleus muscle of TQ-treated compared to control mice. Liver and soleus muscle from mice treated with TQ had enhanced phosphorylated (activated) SIRT-1 in both LFD and HFD groups (Fig 7A–7D). We also analyzed the protein expression levels of other SIRT proteins in the liver, and did not see a difference with TQ treatment across groups for SIRT-2, SIRT-3, SIRT-5, SIRT-6, and SIRT-7 (S1 Fig). This could be due to SIRTs 2–7 having a lower deacetylase activity, as SIRT-6 and SIRT-7 have been previously shown to have a lower NAD^+^-deacetylase activity compared to SIRT-1 [34]. In the liver, TQ enhanced AMPKα phosphorylation as well as phosphorylation of Akt (protein kinase B), a key member of insulin signaling pathway [35,36] (Fig 8A and 8B).

**Fig 9:**
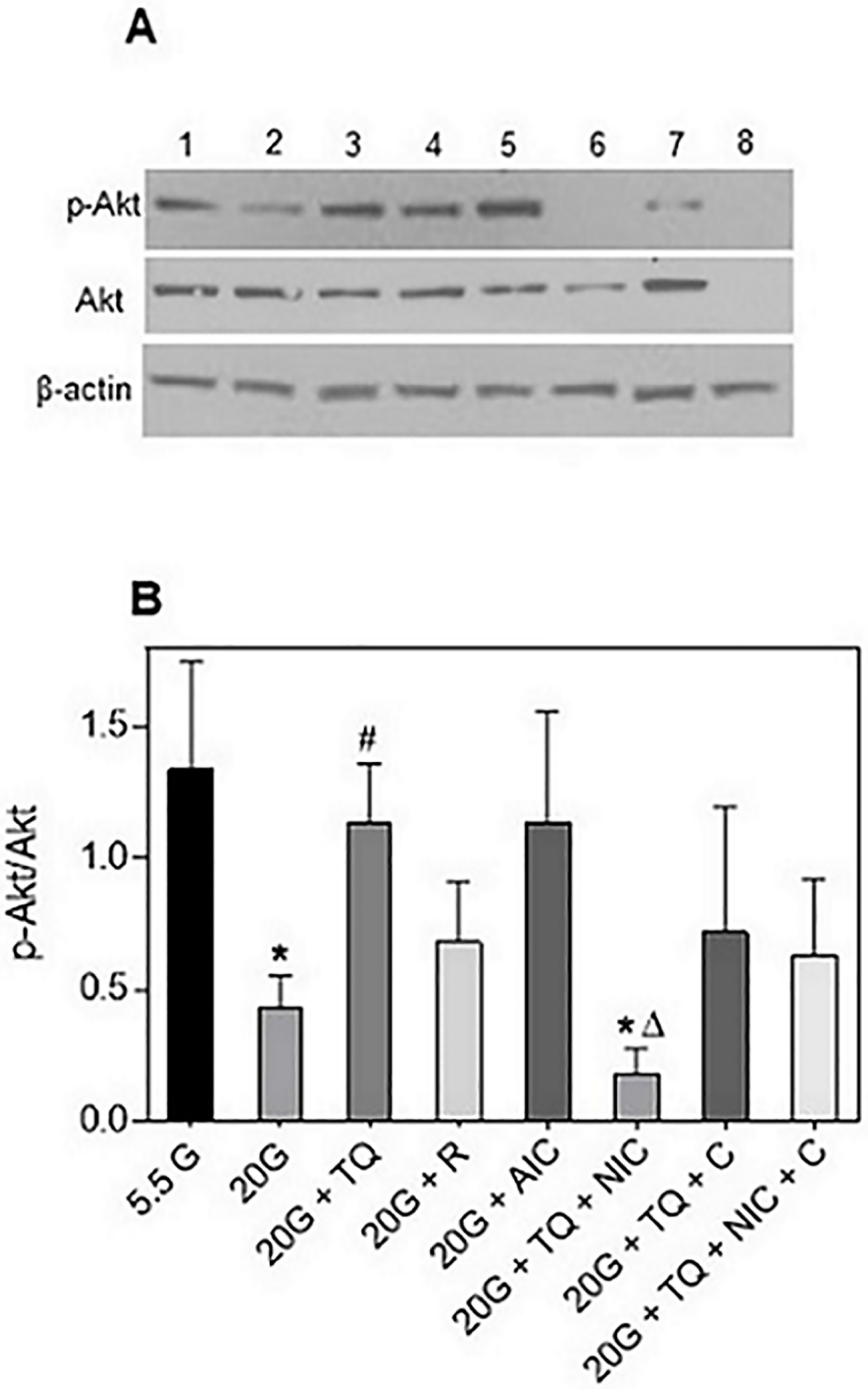
TQ improves insulin sensitivity in HepG2 cells via a SIRT-1 dependent mechanism. HepG2 cells were cultured in high (20 mM) glucose or in growth media containing 5.5 mM glucose for 18 hours, starved with serum-free media for 2 hours, then pre-incubated with vehicle control (0.5% DMSO), nicotinamide (0.5 mM), compound C (20 μM), or with nicotinamide and compound C together for 30 mins, followed by incubation with TQ (10 μM) in the presence or absence of nicotinamide and compound C; or with TQ, resveratrol (50 μM), or AICAR (2 mM) alone for 24 hours in 20mM glucose media. Vehicle-treated cells in 5.5 mM glucose served as control. Insulin (100 nM) was added during the last 30 min. (A) Western blot images of p-Akt, Akt, and β-actin. (B) Protein band quantification using densitometry from three independent experiments. p≤ 0.05 where (*) is significantly different from 5.5G, (#) is significantly different from 20G, and (Δ) is significantly different from 20G + TQ using independent t-tests. 5.5 G: 5.5 mM glucose, 20G: 20 mM glucose, TQ: thymoquinone, R: resveratrol, AIC: AICAR, NIC: nicotinamide, C: compound C.

To evaluate the mechanistic actions behind TQ-induced insulin sensitivity, we used the HepG2 cell line as an *in vitro* model of insulin resistance to assess whether this action is SIRT-1-dependent. HepG2 cells were made insulin resistant as previously described [18, 19], which was confirmed by decreased p-Akt protein after high glucose treatment (Fig 9A and 9B). TQ increased p-Akt in high-glucose treated cells, restoring these levels to that of the control cells (Fig 9A and 9B). This shows that TQ improves insulin resistance in similar fashion to what we see in livers of DIO mice. This action showed to be SIRT-1-dependent, as pre-treatment of insulin resistant cells with SIRT-1 inhibitor nicotinamide in the presence of TQ, significantly decreases p-Akt protein and TQ-induced insulin sensitivity (Fig 9A and 9B). Furthermore, treatment with SIRT-1 activator resveratrol and AMPKα activator AICAR increased insulin sensitivity, although this trend was not statistically significant (Fig 9A and 9B). Pre-treatment with compound C (AMPKα inhibitor) or compound C and nicotinamide in the presence of TQ decreased insulin sensitivity compared to TQ treatment alone, albeit statistically insignificant (Fig 9A and 9B). TQ treatment showed similar trends to the *in vivo* experiments in increasing phosphorylation of SIRT-1 and AMPKα in insulin-resistant cells (S2A–S2D Fig). Trends were also observed in increased p-SIRT-1 and p-AMPKα with resveratrol and AICAR in the presence of TQ (S2A–S2D Fig), as well as a decrease in phosphorylation of SIRT-1 with nicotinamide or compound C in the presence of TQ after high glucose treatment (S2A and S2B Fig). Pre-treatment with compound C or with compound C and nicotinamide significantly decreased p-AMPKα in the presence of TQ compared to TQ treatment alone (S2C and S2D Fig). Taken together, these results provide additional support about the role of TQ in improving insulin resistance, as well as show that this action is likely mediated by SIRT-1 activation.

**Fig 10:**
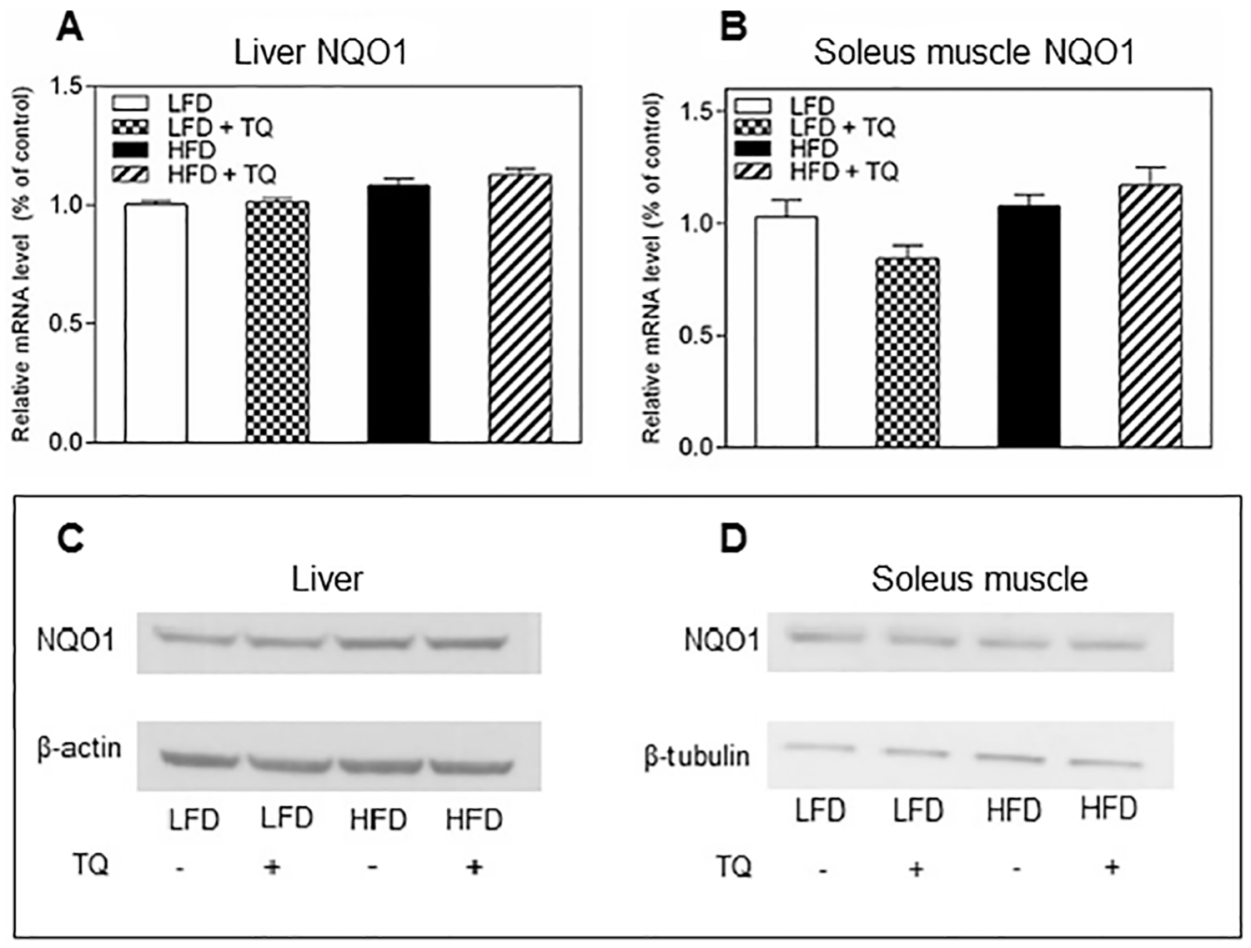
Effects of TQ on NQO1 expression. NQO1 mRNA expression in liver (A) and soleus muscle (B). (C) Western blot images of NQO1 and β-actin protein in liver (D) Western blot images of NQO1 protein in soleus muscle. β-tubulin was used as a loading control. Statistical analysis (A and B): one-way ANOVA followed by Sidak post-test (p≤0.05). qPCR results are means ± SEM (n = 8–12 mice per treatment group). Western blot images are representative of combined liver and soleus muscle lysates from n = 10–12 mice per treatment group. LFD: low fat diet, HFD: high fat diet, TQ: thymoquinone.

TQ applied in this study was within the physiologically relevant diet-derived levels. However, non-physiologically high and toxic levels of quinones is known to generate excessive levels of reactive oxygen intermediates via quinone-dependent redox cycling, and this causes induction of the NAD(P)H-dependent Quinone Oxidoreductase 1 (NQO1). NQO1 is a phase 2 detoxification enzyme induced in response to oxidative stress, which expression is regulated by the Keap1/Nrf2/ARE pathway [10, 37], and NQO1 alone has been show to regulate NADH/NAD^+^ ratio [10, 38]. To ascertain that applied doses of TQ were physiologically low and not inductive of NQO1 and/or oxidative stress, mRNA and protein levels of NQO1 were measured in liver and muscle. Levels of NQO1 were not elevated in any of these tissues, confirming that applied doses, while effective in regulating the cellular redox, do not activate the Keap1/Nrf2/ARE pathway and do not increase oxidative stress (Fig 10). This further supports our hypothesis that TQ-dependent re-oxidation of NADH and consequent decrease of the NADH/NAD^+^ ratio is the main mechanism to activate SIRT-1/AMPK pathway and promote fuel oxidation rather than deposition, which leads to the observed changes in normalization of glucose homeostasis in DIO mice following TQ administration.

## Discussion and conclusions

This is the first *in vivo* study aimed to comprehensively evaluate the effect of thymoquinone (TQ), a bioactive component of the *Nigella sativa* plant, on whole body glucose homeostasis using a physiologically-relevant mouse model of type 2 diabetes. In our published *in vitro* study, we have reported that both *Nigella sativa* extract (NSE) of high thymoquinone (TQ) content, as well as TQ alone, decreased NADH/NAD^+^ ratio and stimulated glucose and fatty acid oxidation in pancreatic β-cells, and this action was accompanied by the restoration of the glucose-stimulated insulin secretion (GSIS) in cells exposed to glucose overload [11]. Here we have expanded our studies to an *in vivo* model with focus on the TQ effect on the insulin sensitive peripheral tissues, and evaluated the action of TQ on glucose homeostasis in Diet Induced Obesity (DIO) mice.

After 24 weeks of HFD, C57/BLJ mice became obese and diabetic, as demonstrated by their increased body weight (Fig 1A), elevated fasting blood glucose (Fig 1B), insulin (Fig 1C) and impaired OGTT and ITT (Fig 2). While TQ treatment improved all these parameters in HFD animals, TQ had no significant effect on weight, fasting blood glucose and insulin, or OGTT /ITT in animals treated with LFD, suggesting that TQ primarily affects DIO metabolism by increasing oxidation of diet-derived fatty acid surplus. However, it is still possible that TQ treatment beyond the 24 weeks could lead to observed changes in physiological parameters in the LFD group as well, and further studies are required to address this issue. Bioavailability of TQ after an oral administration can be a limiting factor on TQ actions. Although such studies have been very limited in mice, studies with other animal models have shown that TQ is rapidly eliminated and slowly absorbed [39,40]. Therefore, further studies are required to address the bioavailability of TQ after oral administration in mice to properly determine a relevant dose and exposure window, particularly in physiologically relevant mouse models of type 2 diabetes.

Metabolism is governed by the oxidation status of nicotinamide adenine dinucleotide, represented by the ratio between its reduced and oxidized forms (NADH/NAD^+^) [14]. During glycolysis NAD^+^ is reduced to NADH, which needs to be re-oxidized back to NAD^+^ [14]. In chronic hyperglycemic conditions, such as in type 2 diabetes, there can be NADH overproduction due to the fact that mitochondrial shuttles are unable to efficiently re-oxidize NADH, which leads to the condition known as reductive stress [14, 41]. This leads to increased pressure on mitochondrial complex I, the primary site of NADH recycling, which in turn causes the formation of superoxide [14, 42] and enhanced oxidative stress, known to be detrimental to insulin sensitivity and insulin secretion and exacerbate the diabetic phenotype [43]. NADH excess inhibits glycolytic and TCA cycle enzymes (glyceraldehyde 3-phosphate dehydrogenase, pyruvate dehydrogenase, isocitrate dehydrogenase, α-ketoglutarate dehydrogenase, malate dehydrogenase), leading to the impairment of glucose oxidation and TCA cycle oxidative pathways [14, 43]. TQ has been shown to regulate oxidation level of adenine nucleotides [11]. Due to its conjugated double bond system, TQ is able to re-oxidize NADH in the process of NAD(P)-dependent redox cycling [44], and thus decrease the NADH/NAD^+^ ratio, as shown by our group [11]. Furthermore, in this study we also demonstrate that TQ treatment leads to a decrease in the NADH/NAD^+^ ratio in liver and skeletal muscle in HFD mice (Fig 6). Regeneration of NAD^+^ from TQ can thus increase glucose and fatty acid oxidation and ameliorate diabetic dyslipidemia. Diabetic dyslipidemia is characterized by high plasma triglyceride concentration, low HDL cholesterol and elevated non-HDL cholesterol [33]. The main cause of this phenotype is the increased free fatty acid release from insulin-resistant adipose tissue in type 2 diabetes [45, 46]. The influx of free fatty acids in the liver can promote triglyceride synthesis, which increases the production of non-HDL (LDL, VLDL) cholesterol to transfer lipids to tissues and decreases HDL cholesterol levels, which transfers lipids back to liver for degradation [33]. Indeed, our data demonstrating that TQ treatment decreased serum LDL/VLD levels (while not affecting HDL levels) and tissue level of triglycerides (Figs 4 and 5) in HFD mice are consistent with TQ antidiabetic action and effect on lipid homeostasis. TQ-dependent decrease in triglyceride and LDL/VLDL levels correlated with improved insulin signaling and insulin sensitivity judged by enhanced phosphorylation of Akt (Fig 8). Akt activation is consistent with the observed improvement in insulin sensitivity seen with the insulin tolerance test (Fig 2B). These results are in accordance with our previously reported *in vitro* results [11] that TQ increases glucose and fatty acid oxidation, which can lead to enhanced fuel oxidation by peripheral tissues, weight loss and increased insulin sensitivity.

In addition to serving as a regulator of metabolic flux and substrate for metabolic processes, NAD^+^ can activate sirtuin 1 (SIRT-1) and consequently SIRT-1-dependent pathways [15]. SIRT-1 is a class III histone deacetylase, where NAD^+^ functions as a substrate for SIRT-1 deacetylation of target proteins [15]. SIRT-1 has been implicated directly in critical aspects of glucose homeostasis, such as increasing insulin secretion and insulin sensitivity, and lowering the inflammation and oxidative stress associated with diabetes and obesity [15, 47–49]. Enhanced production of NAD^+^ via TQ-dependent redox cycling is consistent with increased level of SIRT-1 phosphorylation in liver and muscle (Fig 7A–7D). It has been previously shown that SIRT-1 can activate AMPK (AMP-activated protein kinase) by de-acetylating and activating serine-threonine liver kinase B1 (LBK1), an upstream activator of AMPK [50]. AMPK is activated when cellular energy levels are low (e.g. high AMP/ATP ratio), and has been shown to enhance fatty acid oxidation, glycolysis, stimulate glucose uptake in skeletal muscle, and inhibit cholesterol synthesis [51]. We saw increased phosphorylated AMPKα protein in the liver of both LFD and HFD animals treated with TQ (Fig 8), suggesting that TQ can activate AMPK-dependent pathways. Due to similarities in their action on different processes, such as cellular metabolism and inflammation, it has been suggested that AMPK and SIRT-1 are involved in a cycle where they regulate each other [50]. Whether TQ administration activates AMPK indirectly via SIRT-1, or directly via alteration of parameters different from NADH/NAD^+^ ratio, warrants further investigation. To mechanistically explore whether the increase in insulin sensitivity with TQ treatment is SIRT-1-dependent, we used the HepG2 cell line as a model of insulin resistance. TQ treatment reversed insulin resistance after 24 hours, shown by the increase in phosphorylated Akt (Fig 9). Pre-treatment with SIRT-1 inhibitor nicotinamide suppressed this TQ effect on insulin signaling, suggesting that it is likely SIRT-1-dependent. Pre-treatment with AMPKα inhibitor compound C also inhibited the effect of TQ, albeit statistically insignificant. Furthermore, there was an improvement in insulin resistance after treatment with SIRT-1 and AMPK activators, suggesting a positive role of these pathways in insulin signaling.

Diabetes and obesity are associated with tissue inflammation, which can exacerbate insulin resistance. Adipose-derived pro-inflammatory markers such as resistin and chemokines (MCP-1) can exacerbate insulin resistance by activating c-Jun N-terminal (JNK) kinases and NF-κB transcription factors, which can promote serine phosphorylation (inhibition) of insulin receptor substrate-1 (IRS-1), a key component of insulin signaling [52]. SIRT-1 has been shown to inhibit NF-κB activity, and therefore suppress the inflammatory process [53]. Indeed, TQ treatment decreased serum levels of the pro-inflammatory marker resistin (Fig 3A). Lower resistin levels are consistent with observed increase in the insulin sensitivity in HFD animals (Fig 2B). Since resistin has been shown to increase LDL levels [54], lowering of this marker is also consistent with the observed decreases in serum LDL cholesterol (Fig 5C).

Taken together, our study shows that TQ administration improves glucose tolerance and insulin sensitivity in the diet-induced obesity (DIO) mouse model of type 2 diabetes. Furthermore, TQ treatment has the potential to ameliorate inflammation, altered lipid profile, and weight gain associated with the diabetic and obese state. These anti-diabetic effects of TQ may be mediated by activating SIRT-1 and AMPK pathways, as shown from this study. Our results add to the existing evidence supporting the role of TQ as a natural therapeutic for the treatment of type 2 diabetes, however, further studies are necessary to establish the potential of TQ to treat type 2 diabetes in humans.

## Supporting information

S1 Fig Effect of TQ on expression of other SIRT proteins. Western blot images showing protein expression of SIRT-2, SIRT-3, SIRT-5, SIRT-6, SIRT-7, and β-actin in liver. LFD: low fat diet, HFD: high fat diet, TQ: thymoquinone.
(TIF)

S2 Fig Effect of TQ on SIRT-1 and AMPKα activation in HepG2 cells. HepG2 cells were cultured in high (20 mM) glucose or in growth media containing 5.5 mM glucose for 18 hours, starved with serum-free media for 2 hours, then pre-incubated with vehicle control (0.5% DMSO), nicotinamide (0.5 MM), compound C (20 μM), or with nicotinamide and compound C together for 30 mins, followed by incubation with TQ (10 μM) in the presence or absence of nicotinamide and compound C; or with TQ, resveratrol (50 μM), or AICAR (2 mM) alone for 24 hours in 20mM glucose media. Vehicle-treated cells in 5.5 mM glucose served as control. Insulin (100 nM) was added during the last 30 min. (A) Western blot images of p-SIRT-1, SIRT-1, and β-actin. (C) Western blot images of p-AMPKα, AMPKα, and β-actin. (B and D) Protein band quantification using densitometry from three independent experiments. p≤ 0.05 where (*) is significantly different from 20G + TQ using independent t-tests. 5.5 G: 5.5 mM glucose, 20G: 20 mM glucose, TQ: thymoquinone, R: resveratrol, AIC: AICAR, NIC: nicotinamide, C: compound C.
(TIF)

S1 File Data for Fig 1. GraphPad file with corresponding data for Fig 1.
(PZFX)

S2 File Data for Fig 2. GraphPad file with corresponding data for Fig 2.
(PZFX)

S3 File Data for Fig 3. GraphPad file with corresponding data for Fig 3.
(PZFX)

S4 File Data for Fig 4. GraphPad file with corresponding data for Fig 4.
(PZFX)

S5 File Data for Fig 5. GraphPad file with corresponding data for Fig 5.
(PZFX)

S6 File Data for Fig 6. GraphPad file with corresponding data for Fig 6.
(PZFX)

S7 File Data for Figs 7 and 8. GraphPad file with corresponding data for Fig 7 (panels C and D) and Fig 8 (panel B).
(PZFX)

S8 File Data for Fig 9. GraphPad file with corresponding data for Fig 9 (panel B).
(PZFX)

S9 File Data for Fig 10. GraphPad file with corresponding data for Fig 10 (panels A and B).
(PZF)
