# Supplementary figures and images for: Thymoquinone ameliorates diabetic phenotype in Diet-Induced Obesity mice via activation of SIRT-1-dependent pathways
Source: PLoS One. 2017 Sep 26;12(9):e0185374. doi: 10.1371/journal.pone.0185374 (PMC5614580; doi:10.1371/journal.pone.0185374)

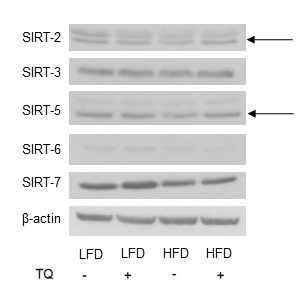

Supplement: S1 Fig — Western blot images showing protein expression of SIRT-2, SIRT-3, SIRT-5, SIRT-6, SIRT-7, and β-actin in liver. LFD: low fat diet, HFD: high fat diet, TQ: thymoquinone. (TIF) [file pone.0185374.s001.tif]

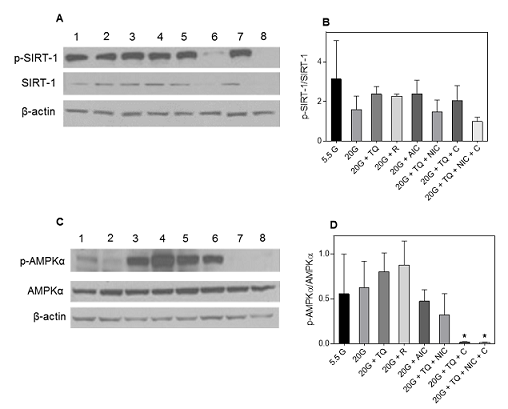

Supplement: S2 Fig — HepG2 cells were cultured in high (20 mM) glucose or in growth media containing 5.5 mM glucose for 18 hours, starved with serum-free media for 2 hours, then pre-incubated with vehicle control (0.5% DMSO), nicotinamide (0.5 MM), compound C (20 μM), or with nicotinamide and compound C together for 30 mins, followed by incubation with TQ (10 μM) in the presence or absence of nicotinamide and compound C; or with TQ, resveratrol (50 μM), or AICAR (2 mM) alone for 24 hours in 20mM glucose media. Vehicle-treated cells in 5.5 mM glucose served as control. Insulin (100 nM) was added during the last 30 min. (A) Western blot images of p-SIRT-1, SIRT-1, and β-actin. (C) Western blot images of p-AMPKα, AMPKα, and β-actin. (B and D) Protein band quantification using densitometry from three independent experiments. p≤ 0.05 where (*) is significantly different from 20G + TQ using independent t-tests. 5.5 G: 5.5 mM glucose, 20G: 20 mM glucose, TQ: thymoquinone, R: resveratrol, AIC: AICAR, NIC: nicotinamide, C: compound C. (TIF) [file pone.0185374.s002.tif]
